# Hematological Diseases and Osteoporosis

**DOI:** 10.3390/ijms21103538

**Published:** 2020-05-16

**Authors:** Agostino Gaudio, Anastasia Xourafa, Rosario Rapisarda, Luca Zanoli, Salvatore Santo Signorelli, Pietro Castellino

**Affiliations:** Department of Clinical and Experimental Medicine, University of Catania, 95123 Catania, Italy; axourafa@gmail.com (A.X.); dott.rapisardaro@tiscali.it (R.R.); dott.zanoli@gmail.com (L.Z.); ssignore@unict.it (S.S.S.); pcastell@unict.it (P.C.)

**Keywords:** osteoporosis, multiple myeloma, monoclonal gammopathy of undetermined significance (MGUS), thalassemia, mastocytosis, hemophilia

## Abstract

Secondary osteoporosis is a common clinical problem faced by bone specialists, with a higher frequency in men than in women. One of several causes of secondary osteoporosis is hematological disease. There are numerous hematological diseases that can have a deleterious impact on bone health. In the literature, there is an abundance of evidence of bone involvement in patients affected by multiple myeloma, systemic mastocytosis, thalassemia, and hemophilia; some skeletal disorders are also reported in sickle cell disease. Recently, monoclonal gammopathy of undetermined significance appears to increase fracture risk, predominantly in male subjects. The pathogenetic mechanisms responsible for these bone loss effects have not yet been completely clarified. Many soluble factors, in particular cytokines that regulate bone metabolism, appear to play an important role. An integrated approach to these hematological diseases, with the help of a bone specialist, could reduce the bone fracture rate and improve the quality of life of these patients.

## 1. Introduction

Despite its inert appearance, bone is a dynamic tissue that is continuously resorbed by osteoclasts and neoformed by osteoblasts. The bone remodeling, which is a highly complex process, is under the control of local and systemic factors that all together contribute to bone homeostasis. Besides osteoclasts and osteoblasts, it has been demonstrated that osteocytes, which comprise 90–95% of the total bone cells, play a key role during the bone remodeling cycle [1]. Osteocytes act as orchestrators producing factors, such as RANKL and sclerostin, that influence osteoclast and osteoblast activities [2]. In the last decade, numerous studies have supported the role of some factors released by osteocytes in the pathogenesis of metabolic bone diseases [3], but also of rheumatological [4] and systemic diseases [5,6].

Osteoporosis is a common skeletal disorder characterized by compromised bone strength that predisposes patients to an increased risk of fracture [7]. A thorough search for underlying causes of the disease reveals that up to 30% of post-menopausal women and between 50 and 80% of men suffer from diseases or have factors contributing to osteoporosis [8,9]. Among the secondary forms of osteoporosis, hematological diseases play a very important role. It seems logical to think that, given the close relationships between bone and bone marrow, alterations in the latter can also have a significant impact on bone health. Studies conducted in animal models showed that bone cells interact with hematopoietic cells, providing a supportive microenvironment needed to maintain erythropoiesis and myelopoiesis [10]. Nevertheless, the effects of hematological diseases on bone are not only caused by the close interconnections between bone marrow cells and bone but are also due to a whole series of circulating factors, such as cytokines, that can alter bone turnover, increasing the activity of osteoclasts and/or reducing the action of osteoblasts. There is mounting evidence that anemia per se, that characterizes several hematological diseases, may also be associated with bone fragility [10]. Among the hypothesized mechanisms of this association, hypoxia seems to play an important role. In fact, hypoxia is a potent stimulator of erythropoietin production that stimulates osteoclast precursors and induces bone loss [11]. Iron deficiency, which is observed in chronic blood loss, may also affect bone health. Iron, in fact, is an essential cofactor for hydroxylation of prolyl and lysil residues of procollagen and participates in vitamin D metabolism through the cytochromes P450 [12]. Finally, bone tissue can be affected by systemic complications related to hematological diseases [9]. Among these pathological conditions, those for which we have more scientific evidence to support a negative effect on bone health are monoclonal gammopathies of undetermined significance and multiple myeloma, systemic mastocytosis, thalassemia major, sickle cell disease, and hemophilia.

## 2. Data Source and Search

A literature search strategy was developed by an experienced team of specialists by consulting the MEDLINE platform. The literature search performed included published papers and reviews updated to December 2019. The search strategy used a combination of controlled key words (e.g., “monoclonal gammopathies of undetermined significance”, “multiple myeloma”, “systemic mastocytosis”, “thalassemia major”, “sickle cell disease”, “hemophilia”, “osteoporosis”, “bone metabolism”, and “fracture”). The search results were limited to papers published in English.

## 3. Monoclonal Gammopathy of Undetermined Significance

Monoclonal gammopathy of undetermined significance (MGUS) is a plasma cell disorder characterized by increased production of an abnormal monoclonal paraprotein of which serum levels are less than 30 g/L, infiltration of the bone marrow by a clonal population of plasma cells less than 10% but without any evidence of end-organ involvement. MGUS is a common condition especially in the elderly, and the risk of progression to multiple myeloma is approximately 1% per year [13].

### 3.1. Bone Involvement

Among its different recognized complications, MGUS appears to also be associated with bone health outcomes. In fact, MGUS patients have neither lytic lesions in the bone nor hypercalcemia, but they present a greater risk of developing osteoporosis and vertebral and hip fractures [14,15,16,17]. One study described that 53.8% of patients with MGUS were osteopenic and 26.2% had osteoporosis, and those with lower bone mineral density were more likely to have had vertebral fractures. Fracture risk in these patients does not depend on the immunoglobulin class of MGUS nor on the concentration of paraprotein, suggesting that all patients with MGUS have an increased risk of fracture [18]. Studies employing high-resolution peripheral quantitative computed tomography measurement of radium showed an increase in bone size with greater cortical porosity and reduced cortical and trabecular thickness and, therefore, reduced bone strength [19,20]. In addition, other studies have highlighted the presence of high levels of bone markers (osteocalcin, alkaline phosphatase, and others) in patients with MGUS, indicating abnormal bone turnover [21,22]. In a study conducted by Thorsteinsdottir et al. [23], the risk of fractures was not significantly increased in individuals with MGUS (HR, 1.19; 95% CI, 0.94–1.50), but when patients were divided according to gender, men with MGUS had a significantly increased risk of fractures, compared with those men without MGUS (HR, 1.49; 95% CI, 1.03–2.08). No increased risk of fractures was found in women with MGUS compared with those without MGUS (HR, 1.02; 95% CI, 0.74–1.40). The reasons for this increased risk of fractures, especially in male subjects with MGUS, are yet to be elucidated. However, MGUS seems to be associated with uncoupling of bone turnover, with increased bone resorption and reduced bone formation due to a higher production of proinflammatory and bone resorptive cytokines [24]; moreover, female sex hormones could possibly play a protective role.

### 3.2. Treatment

Patients affected by MGUS might benefit from therapy to prevent osteoporosis and fractures. The use of bisphosphonates (alendronate or zoledronate) in MGUS patients is effective in improving bone mineral density (BMD), but there is no clear evidence for a reduction of skeletal fracture risk [25,26]. In these subjects, calcium, and vitamin D supplementation is mandatory. It is important to monitor serum calcium levels, because the progression of MGUS to multiple myeloma, although rare, may occur [27].

## 4. Multiple Myeloma

Multiple myeloma (MM) is a frequent hematological malignancy in the elderly with an annual mortality rate of 4.1/100,000 [28]. It is characterized by infiltration of the bone marrow by clonally transformed plasma cells (≥10%) and the subsequent production of monoclonal proteins in the blood and/or urine also accompanied by organ damage. Active myeloma is defined by the presence of one or more of the CRAB criteria: serum calcium elevation (calcium >11 mg/dL), renal dysfunction (creatinine >2 mg/dL), anemia (hemoglobin <10 mg/dL), and bone disease (one or more osteolytic lesions) [29]. In the absence of CRAB features, one or more of the following conditions is required: clonal bone marrow plasma cell percentage ≥60%, serum free light chain ratio ≥100 and ≥1 focal lesions on magnetic resonance studies. Premalignant stages of MM, including MGUS and smoldering myeloma, lack these symptoms and signs [29].

### 4.1. Bone Involvement

Bone disease often occurs during MM. It can cause osteolytic lesions and osteopenia or osteoporosis in about 80% of cases. The most affected skeletal site is the axial skeleton, in particular the vertebral bodies (49%), skull (35%), pelvis (34%), and ribs (33% of patients) [30]. The development of osteolytic bone disease is one of the most important features that typically correlates with progression of the disease from a premalignant state into active MM [31]. Bone disease in myeloma (MBD) can lead to serious complications such as fractures, spinal cord compression, and hypercalcemia, resulting in reduced quality of life due to severe pain, psychological disorders, loss of autonomy, and a significant increase in mortality [30,32].

**Figure 1:**
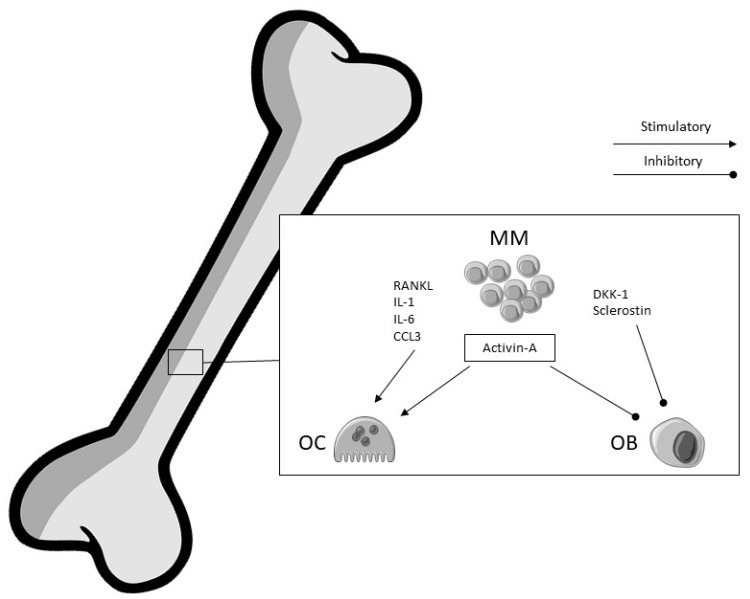
Simplified pathogenesis of osteoporosis in multiple myeloma. MM: multiple myeloma cells; OC: osteoclast; OB: osteoblast; RANKL: receptor activator of NF-κB ligand; IL-1: interleukin-1; IL-6: interleukin-6; CCL3: chemokine C–C motif ligand 3; DKK-1: dickkopf-1.

The pathogenesis of lytic lesions and progressive bone mass loss in multiple myeloma is due to the imbalance of bone remodeling, determined by the activation of osteoclastogenesis and the simultaneous inhibition of osteoblastogenesis including the increase in osteocyte apoptosis. MM cells interfere with physiologic bone remodeling by secreting different cytokines such as receptor activator of NF-κ B ligand (RANKL), interleukin (IL)-1, IL-6, and chemokine C–C motif ligand 3 (CCL3) that promote osteoclast proliferation and activity. MM cells can also upregulate the osteoblast inhibitors dickkopf-1 (DKK1) and sclerostin, thereby inhibiting osteoblastogenesis [33,34,35,36,37]. Moreover, MM can determine the deregulation of the bone compartment, creating a permissive microenvironment for MM cell expansion [38]. To date, it is known that bone marrow stromal cells (BMSCs) can interact with MM cells via adhesion molecules, thus promoting MM cell retention within the bone marrow and contributing to increased osteolytic bone lesions [39]. Both MM and BMSCs cells, apart from the above-mentioned cytokines, can also secrete other factors like vascular endothelial growth factor (VEGF), insulin-like growth factor (IGF-1), TGF-β, angiopoietin-1 (Ang-1), platelet-derived growth factor (PDGF), and basic-fibroblast growth factor (bFGF) [40,41], which have been seen to be associated with increased angiogenesis, osteoclastogenesis, and tumor growth in MM [42]. Additionally, mesenchymal stem cells in MM patients with advanced bone disease have been found to produce activin A, possibly due to an intrinsic genetic defect in BMSCs of these patients. Activin A is an osteoclast-activating factor and an inhibitor of osteoblast differentiation. In MM patients, a correlation between elevated serum levels of activin A and lytic bone lesions has been found (Figure 1) [43,44].

### 4.2. Treatment

MBD treatment aims to reduce the complications and occurrence of related skeletal events (SRE). The use of bisphosphonates, known anticatabolic agents, especially pamidronate and zoledronic acid, given monthly intravenously, is recommended in all patients with symptomatic MM in order to prevent pathological vertebral fractures or other SREs and relieve pain due to bone disease [45,46]. Moreover, bisphosphonates may also have anti-tumor and immunomodulatory activity [47], and clearly they can be used to treat osteoporosis in low- and intermediate-risk asymptomatic MM. A recent meta-analysis found no evidence of superiority of any specific bisphosphonate (amino or non-amino) for any outcome. However, zoledronate was found to be better than placebo and etidronate for improving overall survival and reducing vertebral fractures [48], and it has been seen to be more effective than pamidronate for the treatment of hypercalcemia of malignancy [49]. Denosumab, a human anti-RANKL monoclonal antibody, given subcutaneously, has also proven to be an effective treatment strategy in MM. It binds with high affinity and specificity to RANKL and inhibits bone resorption [50]. The efficacy of denosumab compared to zoledronate was evaluated in a large, randomized clinical trial that showed no difference between the two types of treatment [51]. Unlike zoledronate, denosumab can be administered in patients with impaired renal function, which is very important given that kidney dysfunction is common in MM and often represents a limit to the use of bisphosphonates. However, there are no recommendations regarding treatment duration, while caution is advised in case of interruption, since denosumab has a rebound effect [52].

New anti-cancer therapeutic agents for MM that also have effects on bone metabolism are proteasome inhibitors, such as bortezomib and carfilzomib, and Wnt system modulators like DKK1 and sclerostin antagonists. In the first group, bortezomib, by inhibiting the proteasome, stimulates the activity and number of osteoblasts with a consequent increase in bone formation and simultaneously inhibits osteoclastogenesis and osteoclast bone resorption activity [53,54]. In addition, bortezomib partially inhibits osteocyte autophagy and apoptosis induced by MM and consequently increases the number of viable osteocytes [54]. Other possible therapeutic options include BHQ880 and romosozumab. The first one is an anti-DKK1 IgG1 antibody, which stimulates osteoblast differentiation and inhibits myeloma cell growth via alteration of the bone marrow microenvironment [55,56]. BHQ880 in combination with zoledronate and anti-myeloma treatments was well-tolerated and increased bone mineral density in patients with relapsing or refractory MM [57]. Romosozumab, on the other hand, is a humanized anti-sclerostin monoclonal antibody, which has been seen to be useful, in preclinical studies in MM, in preventing the development of MBD in the early stages of the disease and in reducing osteolysis by increasing bone mass in advanced MM [58,59]. Since sclerostin inhibition does not have significant antitumor activity, combination strategies can be essential to achieve anti-MM and bone-protective effects.

Since sclerostin is produced by osteocytes, romosozumab may be a more effective therapeutic strategy. On the contrary, as DKK1 is mainly produced in MM by malignant plasma cells and not all patients express it, there could be individual differences in response to anti-DKK1 therapies [33].

Ongoing research primarily revolves around agents that stimulate bone formation by restoring balanced bone turnover, thus improving BMD, slowing down disease progression and restoring bone damage.

## 5. Systemic Mastocytosis

Systemic mastocytosis (SM) is a hematological disease characterized by neoplastic proliferation of abnormal mast cells and their accumulation in various organs other than skin (bone marrow, bones, spleen, lymph nodes, and the gastrointestinal tract) [60]. It represents the most aggressive form of mastocytosis, mainly affecting adult subjects, and it may be associated with multiorgan dysfunction and reduced survival [61], whereas cutaneous mastocytosis is more frequent in children and consists of skin-limited disease without systemic involvement and a good prognosis [62]. In most cases, SM is caused by a mutation in the gene that codes for the KIT receptor, a transmembrane receptor with tyrosine-kinase activity expressed by mast cells. The interaction between KIT and its ligand, stem cell factor (SCF), plays an important role in the regulation of proliferation, maturation, adhesion, chemotaxis, and survival of mast cells [63]. The WHO classification [60] includes SM between chronic myeloproliferative neoplasms and describes various forms with a heterogeneous clinical presentation and prognosis. To make the diagnosis of SM, a major criterion and one minor criterion, or three minor criteria must be satisfied (Table 1). In the diagnosis of SM, the measurement of serum tryptase appears to be a good screening tool, though bone marrow biopsy should be considered if there is suspicion of false positives and in patients with normal serum tryptase if there are other clinical signs that suggest SM. Among clinical manifestations, anaphylaxis is the most severe and can be triggered by physical stimuli, emotional factors, drugs, alcohol, surgery, and others [60].

### 5.1. Bone Involvement

In adults, SM frequently causes bone involvement: osteopenia and/or osteoporosis, bone fractures, osteolytic and/or sclerotic lesions, and diffuse osteosclerosis. In recent years, numerous epidemiological studies have been published on bone involvement in SM [64,65,66]. Many of these included elderly patients, which could be a confounding factor of the prevalence estimate. Others, instead considering only the clinically apparent fractures, underestimate the results. However, most studies have shown that osteoporosis is the most frequent bone abnormality in patients with SM, and its prevalence varies from 10 to 38% [67]. The data on vertebral fractures are significantly more prevalent in men than in women (20 vs. 14%) [64]. Another bone alteration that can occur in SM is local or diffuse osteosclerosis, which has a prevalence ranging from 5.3 to 10% [67]. Its prevalence is probably underestimated due to the absence of symptoms and the need for skeletal X-rays to make a diagnosis. Furthermore, in SM patients, focal osteosclerotic and osteolytic bone lesions can often coexist [67]. In these patients a higher bone turnover has been found in both osteoporosis and osteosclerosis [68]; therefore, fractures can be associated with both of these disorders. In addition, the level of tryptase may predict mast cell mass and simultaneously best correlate to higher bone density; however, this high bone density does not protect against fracture in SM patients [69].

The reduction in bone mass mainly concerns trabecular rather than cortical bone, as demonstrated by the greater prevalence of osteoporosis and vertebral fractures relative to non-vertebral ones. This appears to be due to the infiltration of mast cells in the bone marrow and the local release of mediators like histamine, heparin, tryptase, tumor necrosis factor, IL-1, IL-17, and IL-6, with effects both on osteoblasts and osteoclasts [70,71,72]. Once produced by mast cells, histamine, for example, is capable of increasing bone resorption, both directly by stimulating the differentiation and activation of osteoclasts and their precursors and indirectly by increasing the expression of RANKL in osteoblasts [73].

The bone pathophysiology in SM is very complex, and the factors that determine a low BMD rather than a form of osteosclerosis or both are still poorly understood. However elevated bone levels of both RANKL and osteoprotegerin (OPG) have been found in mastocytosis [74,75]. It is thought that tryptase produced by mastocytes could activate osteoblasts and increase the production of OPG, thereby increasing bone turnover and formation. For this reason, interferon α, which would be able to reduce inflammation and consequently the secretion of tryptase, could also be used to treat osteoporosis in mastocytosis as a complementary therapy to bisphosphonates [76]. On the other hand, in a study conducted by Rossini et al. [74], the authors found that patients with mastocytosis had elevated serum DKK1 levels when compared with controls, which correlated positively with CTX levels and increased bone resorption, while Rabenhorst highlighted elevated sclerostin levels in patients with mastocytosis and bone disease [75]. It is therefore reasonable to think that the RANK/RANKL/OPG system and the Wnt-signaling pathway are involved in the pathogenesis and prevalence of one with respect to the other, determining the phenotypic alteration of bone manifestations [67,77].

### 5.2. Treatment

The therapeutic approach to mastocytosis and osteoporosis is based on the integration of vitamin D and the use of antiresorptive drugs such as bisphosphonates. Bisphosphonates, both oral and parenteral, have a positive effect on the lumbar spine BMD but much less on the femoral one [78,79].

The infusion of zoledronate 5 mg once a year resulted in an average increase in lumbar and femoral BMD [80]. Alpha interferon therapy administered in combination with bisphosphonates has been shown to improve bone density, reduce pain, and prevent further fractures [76]. Perhaps the reduction of inflammation could lead to stability or even to the improvement of bone disease.

Since mast cells have been found to produce RANKL [75,81], the monoclonal anti-RANKL antibody denosumab could be used in patients’ refractory or intolerant to bisphosphonates with positive results for BMD [82]. On the contrary, teriparatide being an anabolic agent indicates some safety problems due to possible growth and proliferation of abnormal mast cells and possible induction of more aggressive forms of SM. For these reasons, its use is not recommended in mastocytosis. More longitudinal data are needed for a better understanding of the evolution of bone involvement and the impact of treatments in SM.

## 6. Thalassemia Major

Thalassemia major (TM) is a hereditary blood disorder characterized by decreased hemoglobin production caused by defective globin synthesis. Common symptoms of the disease are anemia, splenomegaly, and cranial and facial bone enlargement. These bone deformities are due to marked expansion of the bone marrow secondary to anemia and ineffective erythropoiesis [83,84].

### 6.1. Bone Involvement

Nevertheless, along with the improvement of transfusion and chelating regimens, thalassemic patients are frequently affected by osteoporosis and osteopenia [85,86,87]. The prevalence of fractures in TM patients ranges from 16 to 49%, depending on the study population and method of data collection [88,89,90]. Vertebral fractures are usually asymptomatic and underestimated, and their prevalence varies from 2.6 to 13% [91,92].

**Figure 2:**
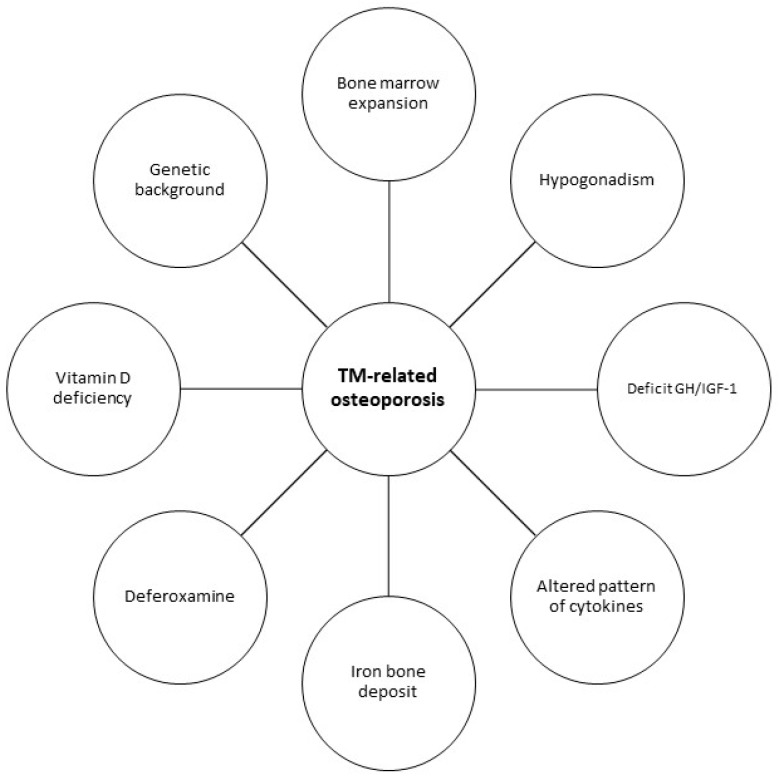
Pathogenetic factors of TM-related osteoporosis. TM: Thalassemia Major.

The pathogenesis of bone loss in TM is not yet fully clarified [87]. Multiple factors can play a role in bone involvement in these patients: bone marrow expansion, hypogonadism, altered pattern of cytokines, deficit GH-IGF-1, iron bone deposit, deferoxamine bone toxicity, genetic background, and vitamin D deficiency [93,94,95,96,97,98,99,100]. All of these pathogenic factors can cause impaired bone turnover, directly and/or indirectly, depressing bone formation and increasing bone resorption (Figure 2).

The mechanism responsible for osteoclast activation in thalassemic patients could be related to the altered cytokine network and in particular to alteration of the RANK/RANKL/OPG system, as we observed in our previous study [101]. Recently, the Wnt pathway has been proposed to participate in the pathogenesis of osteoporosis in TM, and negative modulators of this signaling system, such as DKK-1 and sclerostin, have been also associated with BMD in TM patients [102]. One of the most important factors determining bone destruction in thalassemic patients is represented by bone marrow expansion. In fact, ineffective erythropoiesis despite current regular transfusion regimens is only partially suppressed in these patients. Nowadays, it is known that bone loss in TM largely involves the trabecular bone, and this fact could be due to the close interaction between bone marrow and bone remodeling. Therefore, in TM patients, the most affected site is the lumbar spine due to its constitution of mainly trabecular bone [87].

### 6.2. Treatment

The management of TM to date based on appropriate transfusion and chelating regimens has been successful in extending life expectancy, decreasing comorbidities and improving quality of life.

Chelation therapy is used in order to prevent iron overload in organs like the liver, heart, and endocrine glands [103]. For many years, deferoxamine has been used subcutaneously to treat iron overload, but since its chelating action is not entirely specific for iron, new oral chelating agents have been developed. In addition, hormone replacement therapy can be used to correct hypogonadism since it causes severe bone mass loss in patients with TM [104,105]. Bone marrow transplantation seems to be a very promising option [106]. Regarding TM-related osteoporosis, it is important to ensure that patients have an adequate intake of calcium, vitamin D in particular, and regular physical activity [107]. There are some experiences with different bisphosphonate regimens (clodronate, alendronate, zoledronate, and neridronate), followed in general by an increase in BMD, in particular zoledronate, but without evidence of a fracture rate reduction [108,109].

More recently, Voskaridou et al. [110] observed that Denosumab 60 mg every 6 months, administered subcutaneously in a group of transfusion-dependent thalassemic patients, determined a significant increase in lumbar BMD compared to placebo, with a reduction of bone resorption markers and pain scores, but did not modify femoral BMD. Regarding other therapies, there is limited evidence of a partial effect of strontium ranelate and teriparatide on vertebral BMD [111,112].

## 7. Sickle Cell Disease

Sickle cell disease (SCD) is an inherited blood disorder caused by a single amino acid substitution in the β-globin chain that results in the production of the characteristic Hemoglobin S (HbS). When HbS is deoxygenated, red blood cells are deformed, assuming the typical sickle shape from which the name of the disease derives. SCD is the most important blood disorder over the world for prevalence and social impact, affecting about 300,000 new-borns every year, especially in Sub-Saharan African regions [113].

### 7.1. Bone Involvement

The prevalence of osteopenia and osteoporosis in young adults with SCD is extremely high and exceeded 60% in several studies [114,115]. The most affected sites are: the lumbar spine (55%), the radius (30%), and the femoral neck (15%) [115].

Bone loss in SCS depends on different factors: growth retardation, delayed puberty, vitamin D insufficiency, low physical activity, malnutrition, and release of inflammatory cytokines [116].

It is also possible to observe high-BMD values in SCD patients, especially in those with the S/beta-thalassemia genotypes [117]. It has been observed that osteosclerosis patients had multiple infarctions in the studied bones, due to vaso-occlusive episodes, that led to reduced osteoclast activity and increased BMD. The RANK/RANKL/OPG system could undoubtedly participate in these processes [118]. Also, sex steroids, in particular a deficit of estradiol both in male and female subjects, could contribute to the bone damage [119].

### 7.2. Treatment

To date, there are no specific studies concerning the treatment of osteoporosis/osteopenia in patients with SCD, but since vitamin D deficiency is very common [120], supplementation is reasonable as in the general population.

## 8. Hemophilia

Hemophilia is a rare X-linked inherited bleeding disorder, characterized by a deficiency of coagulation factor (F) VIII (FVIII) (hemophilia A, 85% of cases) or IX (FIX) (hemophilia B) in plasma [121]. FVIII, also known as anti-hemophiliac factor, plays an essential role in the process of blood clotting. In particular, once active, FVIIIa acts as a necessary cofactor for FIXa, which in the presence of Ca2+ and phospholipids forms a complex that converts FX to its active form, FXa. The latter catalyzes the conversion of prothrombin to thrombin, resulting in clot formation [122]. For this reason, affected individuals experience uncontrolled bleeding, both spontaneously or after trauma or surgery, which typically occurs into joints (hemarthrosis) and muscles, but any site can be interested [123]. Although the spectrum of bleeding manifestations can be variable, from superficial ecchymosis to lethal hemorrhage in the central nervous system [124], the phenotype is primarily determined by the residual amount of the deficient coagulation factor, with severe disease defined as <1%, moderate with 1–5% and mild hemophilia with 6–40% FVIII or FIX [122]. The current standard of care for the treatment of bleeding episodes and prophylaxis regimens is replacement therapy using plasma derived or recombinant coagulation factor concentrates [124].

### 8.1. Bone Involvement

In patients with hemophilia (PWH), low BMD or osteoporosis is frequently observed, although the exact pathogenetic mechanisms are not completely elucidated [122]. In fact, 27% of PWH have osteoporosis and 43% have low bone density [125]. Men with hemophilia A and B exhibit a significant reduction in both lumbar spine and hip BMD, which appears to begin in childhood [122]. The meta-analysis of Iorio et al. [126] in 2010 confirmed the association between severe hemophilia and low BMD, which is also confirmed by a systematic review and meta-analysis published by Paschou et al. in 2014 [121].

In recent years, there are data in the literature that demonstrate an increased rate of bone resorption among patients with hemophilia [122]. The reasons of this negative effect of hemophilia on bone health are numerous. First of all, hemophilic arthropathy caused by spontaneous intra-articular bleeding leads to structural changes of the joints [127] and the consequent reduction in patients’ physical activity may compromise the acquisition of peak bone mass during childhood and affect BMD in adult life [128]. Blood-borne virus infections, such as hepatitis C virus (HCV) or human immunodeficiency virus (HIV), are highly prevalent in patients with hemophilia and, since they have been associated with low bone mass, they could provide a second pathophysiologic link [125]. Furthermore, 25-hydroxyvitamin D concentrations were shown to be independent predictors of low BMD in men with hemophilia A and B [129]. Over the last few years, some evidence has indicated a novel effect for FVIII outside of the coagulation system. In fact, it may promote bone formation by a thrombin-mediated mitogenic effect on osteoblasts [122].

### 8.2. Treatment

Most of the authors do not recommend routine screening in individuals with hemophilia who are <40 years of age in the absence of a low trauma fracture. Patients with HIV, advanced arthropathy, and/or low BMI should be considered for screening DXA [130]. The only clinical trial for the treatment of low BMD in patients with hemophilia evaluated the effect on BMD of a 12-month-long monthly oral administration of 150 mg Ibandronate, a bisphosphonate, in 10 adults (mean age of 43.5 years) [131]. In this cohort, ibandronate was well tolerated and led to a 4.7% increase in BMD in the lumbar spine, but not to significant changes in the total hip or femoral neck [131]. In PWH, prevention of osteoporosis is of fundamental importance and it should include factor replacement therapy administered on a regular basis to prevent bleeding, a diet adequate in calcium and vitamin D, physical exercise, and limitation of tobacco and alcohol use, as well as limitation of the duration of immobilization [122]. Teriparatide, raloxifene, or bisphosphonates should be avoided to treat osteoporosis in young hemophilics. However, elderly hemophilics may be treated with these drugs, including denosumab [132].

## 9. Conclusions

**Figure 3:**
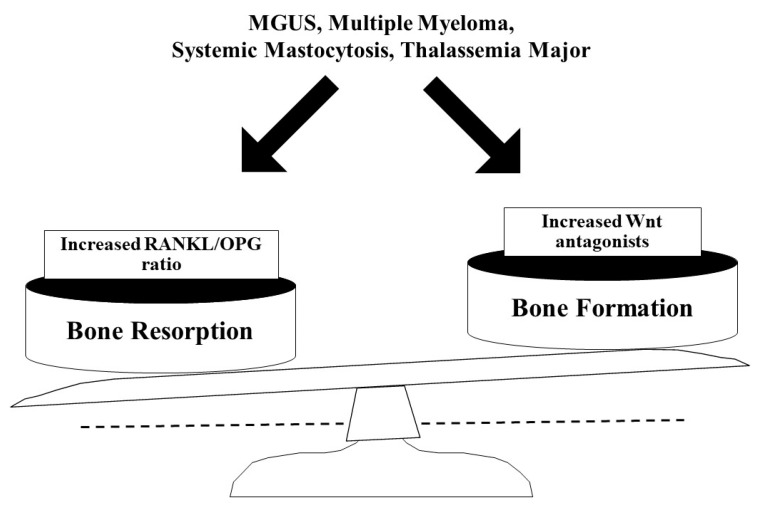
Unbalanced bone turnover in hematological diseases. MGUS: monoclonal gammopathy of undetermined significance; RANKL: receptor activator of NF-κB ligand; OPG: osteoprotegerin.

Hematological diseases represent a frequent cause of secondary osteoporosis. The disease may already be known at the time of anamnestic collection or may be diagnosed thanks to the routine laboratory and clinical investigations to which patients are subjected. Investigations commonly conducted to exclude secondary forms of osteoporosis include, among others, a full blood count, erythrocyte sedimentation rate (ESR), and serum or urine protein electrophoresis, which can help to diagnose paraproteinemia or an anemia. Obviously, other second-level examinations (tryptase, hemoglobin electrophoresis, serum immunofixation, bone marrow biopsy, etc.) and a hematological consultation are necessary to complete the diagnosis. Hematological diseases can determine bone involvement due to direct or indirect mechanisms. In general, their final effect is unbalanced bone turnover with increased bone resorption due to high RANKL levels and reduced bone formation caused by inhibition of Wnt-signaling (Figure 3). These patients should undergo a complete bone check-up, with evaluation of calcium phosphate metabolism, bone turnover markers, vitamin D levels, densitometric exams at the lumbar and femoral levels and, in selected patients, a lateral X-ray of the spine for any asymptomatic vertebral fractures. In general, the therapeutic approach of these patients includes calcium and vitamin D supplementation, and some lifestyle changes, such as the reduction of excessive alcohol intake, smoking cessation, and daily physical activity. Most of the data in the literature supports the use of reabsorption inhibitors such as bisphosphonates in these pathological conditions. Recently, there has also been some evidence of beneficial effects of denosumab. Strict cooperation between the hematologist and bone specialist (internist, endocrinologist, rheumatologist, etc.) is critical to prevent bone fractures in hematologic patients and to ensure a good quality of life.

## Figures and Tables

**Table 1:** Diagnosis of systemic mastocytosis based on World Health Organization criteria.

**Major**
Multifocal, dense aggregates of at least 15 mast cells in the bone marrow (BM) and/or other extracutaneous organ(s)
**Minor**
>25% of mastcells (MC) in the infiltrate of biopsy sections are spindle-shaped or have atypical morphology or, of all MC in the BM aspirate smears, >25% are immature or atypical
Activating point mutation at codon 816 of c-KIT in BM, blood, or another extracutaneous organ
MC in BM, blood, or extracutaneous organs express CD2 and/or CD25 in addition to normal MC markers
Total serum tryptase persistently exceeds 20 ng/mL

## References

[1] Florencio-Silva R., Sasso G.R., Sasso-Cerri E., Simões M.J., Cerri P.S. (2015). Biology of Bone Tissue: Structure, Function, and Factors That Influence Bone Cells. Biomed. Res. Int..

[2] Neve A., Corrado A., Cantatore F.P. (2012). Osteocytes: Central conductors of bone biology in normal and pathological conditions. Acta Physiol..

[3] Gaudio A., Pennisi P., Bratengeier C., Torrisi V., Lindner B., Mangiafico R.A., Pulvirenti I., Hawa G., Tringali G., Fiore C.E. (2010). Increased sclerostin serum levels associated with bone formation and resorption markers in patients with immobilization-induced bone loss. J. Clin. Endocrinol. Metab..

[4] Intemann J., De Gorter D.J.J., Naylor A.J., Dankbar B., Wehmeyer C. (2020). Importance of osteocyte-mediated regulation of bone remodelling in inflammatory bone disease. Swiss Med. Wkly..

[5] Gaudio A., Privitera F., Pulvirenti I., Canzonieri E., Rapisarda R., Fiore C.E. (2014). Relationships between osteoprotegerin, receptor activator of the nuclear factor kB ligand and serum levels and carotid intima-media thickness in patients with type 2 diabetes mellitus. Panminerva Med..

[6] Gaudio A., Fiore V., Rapisarda R., Sidoti M.H., Xourafa A., Catalano A., Tringali G., Zanoli L., Signorelli S.S., Fiore C.E. (2017). Sclerostin is a possible candidate marker of arterial stiffness: Results from a cohort study in Catania. Mol. Med. Rep..

[7] Lorentzon M., Cummings S.R. (2015). Osteoporosis: The evolution of a diagnosis. J. Intern. Med..

[8] NIH (2001). Consensus Development Panel on Osteoporosis Prevention D Therapy. Osteoporosis prevention, diagnosis, and therapy. JAMA.

[9] Mirza F., Canalis E. (2015). Management of endocrine disease: Secondary osteoporosis: Pathophysiology and management. Eur. J. Endocrinol..

[10] Valderrábano R.J., Wu J.Y. (2019). Bone and blood interactions in human health and disease. Bone.

[11] Hiram-Bab S., Liron T., Deshet-Unger N., Mittelman M., Gassmann M., Rauner M., Franke K., Wielockx B., Neumann D., Gabet Y. (2015). Erythropoietin directly stimulates osteoclast precursors and induces bone loss. FASEB J..

[12] Toxqui L., Vaquero M.P. (2015). Chronic iron deficiency as an emerging risk factor for osteoporosis: A hypothesis. Nutrients.

[13] Kyle R.A., Durie B.G., Rajkumar S.V., Landgren O., Blade J., Merlini G., Kröger N., Einsele H., Vesole D.H., Dimopoulos M. (2010). International Myeloma Working Group. Monoclonal gammopathy of undetermined significance (MGUS) and smoldering (asymptomatic) multiple myeloma: IMWG consensus perspectives risk factors for progression and guidelines for monitoring and management. Leukemia.

[14] Melton L.J., Rajkumar S.V., Khosla S., Achenbach S.J., Oberg A.L., Kyle R.A. (2004). Fracture risk in monoclonal gammopathy of undetermined significance. J. Bone Miner. Res..

[15] Bida J.P., Kyle R.A., Therneau T.M., Melton L.J., Plevak M.F., Larson D.R., Dispenzieri A., Katzmann J.A., Rajkumar S.V. (2009). Disease associations with monoclonal gammopathy of undetermined significance: A population-based study of 17,398 patients. Mayo Clin. Proc..

[16] Kristinsson S.Y., Tang M., Pfeiffer R.M., Bjorkholm M., Blimark C., Mellqvist U.H., Wahlin A., Turesson I., Landgren O. (2010). Monoclonal gammopathy of undetermined significance and risk of skeletal fractures: A population-based study. Blood.

[17] Piot J.M., Royer M., Schmidt-Tanguy A., Hoppé E., Gardembas M., Bourrée T., Hunault M., François S., Boyer F., Ifrah N. (2015). Factors associated with an increased risk of vertebral fracture in monoclonal gammopathies of undetermined significance. Blood Cancer J..

[18] Pepe J., Petrucci M.T., Nofroni I., Fassino V., Diacinti D., Romagnoli E., Minisola S. (2006). Lumbar bone mineral density as the major factor determining increased prevalence of vertebral fractures in monoclonal gammopathy of undetermined significance. Br. J. Haematol..

[19] Ng A.C., Khosla S., Charatcharoenwitthaya N., Kumar S.K., Achenbach S.J., Holets M.F., McCready L.K., Melton L.J., Kyle R.A., Rajkumar S.V. (2011). Bone microstructural changes revealed by high-resolution peripheral quantitative computed tomography imaging and elevated DKK1 and MIP-1α levels in patients with MGUS. Blood.

[20] Farr J.N., Zhang W., Kumar S.K., Jacques R.M., Ng A.C., McCready L.K., Rajkumar S.V., Drake M.T. (2014). Altered cortical microarchitecture in patients with monoclonal gammopathy of undetermined significance. Blood.

[21] Woitge H.W., Horn E., Keck A.V., Auler B., Seibel M.J., Pecherstorfer M. (2001). Biochemical markers of bone formation in patients with plasma cell dyscrasias and benign osteoporosis. Clin. Chem..

[22] Laroche M., Attal M., Dromer C. (1996). Bone remodelling in monoclonal gammopathies of uncertain significance, symptomatic and non symptomatic myeloma. Clin. Rheumatol..

[23] Thorsteinsdottir S., Lund S.H., Lindqvist E.K., Thordardottir M., Sigurdsson G., Costello R., Burton D., Steingrimsdottir H., Gudnason V., Eiriksdottir G. (2017). Bone disease in monoclonal gammopathy of undetermined significance: Results from a screened population-based study. Blood Adv..

[24] Veronese N., Luchini C., Solmi M., Sergi G., Manzato E., Stubbs B. (2018). Monoclonal gammopathy of undetermined significance and bone health outcomes: A systematic review and exploratory meta-analysis. J. Bone Miner. Metab..

[25] Pepe J., Petrucci M.T., Mascia M.L., Piemonte S., Fassino V., Romagnoli E., Minisola S. (2008). The effects of alendronate treatment in osteoporotic patients affected by monoclonal gammopathy of undetermined significance. Calcif. Tissue Int..

[26] Berenson J.R., Yellin O., Boccia R.V., Flam M., Wong S.F., Batuman O., Moezi M.M., Woytowitz D., Duvivier H., Nassir Y. (2008). Zoledronic acid markedly improves bone mineral density for patients with monoclonal gammopathy of undetermined significance and bone loss. Clin. Cancer Res..

[27] van de Donk N.W., Palumbo A., Johnsen H.E., Engelhardt M., Gay F., Gregersen H., Hajek R., Kleber M., Ludwig H., Morgan G. (2014). The clinical relevance and management of monoclonal gammopathy of undetermined significance and related disorders: Recommendations from the European Myeloma Network. Haematologica.

[28] Moreau P., San Miguel J., Sonneveld P., Mateos M.V., Zamagni E., Avet-Loiseau H., Hajek R., Dimopoulos M.A., Ludwig H., Einsele H. (2017). Multiple myeloma: ESMO Clinical Practice Guidelines for diagnosis, treatment and follow-up. Ann. Oncol..

[29] Rajkumar S.V., Kumar S. (2016). Multiple Myeloma: Diagnosis and Treatment. Mayo Clin. Proc..

[30] Silbermann R., Roodman G.D. (2013). Myeloma bone disease: Pathophysiology and management. J. Bone Oncol..

[31] Greipp P.R., San Miguel J., Durie B.G., Crowley J.J., Barlogie B., Bladé J., Boccadoro M., Child J.A., Avet-Loiseau H., Kyle R.A. (2005). International staging system for multiple myeloma. J. Clin. Oncol..

[32] Zagouri F., Kastritis E., Zomas A., Terpos E., Katodritou E., Symeonidis A., Delimpasi S., Pouli A., Vassilakopoulos T.P., Michalis E. (2017). Hypercalcemia remains an adverse prognostic factor for newly diagnosed multiple myeloma patients in the era of novel antimyeloma therapies. Eur. J. Haematol..

[33] Vallet S., Filzmoser J.M., Pecherstorfer M., Podar K. (2018). Myeloma Bone Disease: Update on Pathogenesis and Novel Treatment Strategies. Pharmaceutics.

[34] Sezer O., Heider U., Zavrski I., Kühne C.A., Hofbauer L.C. (2003). RANK ligand and osteoprotegerin in myeloma bone disease. Blood.

[35] Giuliani N., Lisignoli G., Colla S., Lazzaretti M., Storti P., Mancini C., Bonomini S., Manferdini C., Codeluppi K., Facchini A. (2008). CC-chemokine ligand 20/macrophage inflammatory protein-3α and CC-chemokine receptor 6 are overexpressed in myeloma microenvironment related to osteolytic bone lesions. Cancer Res..

[36] Udagawa N., Takahashi N., Katagiri T., Tamura T., Wada S., Findlay D.M., Martin T.J., Hirota H., Taga T., Kishimoto T. (1995). Interleukin (IL)-6 induction of osteoclast differentiation depends on IL-6 receptors expressed on osteoblastic cells but not on osteoclast progenitors. J. Exp. Med..

[37] Spaan I., Raymakers R.A., van de Stolpe A., Peperzak V. (2018). Wnt signaling in multiple myeloma: A central player in disease with therapeutic potential. J. Hematol. Oncol..

[38] Podar K., Chauhan D., Anderson K.C. (2009). Bone marrow microenvironment and the identification of new targets for myeloma therapy. Leukemia.

[39] Michigami T., Shimizu N., Williams P.J., Niewolna M., Dallas S.L., Mundy G.R., Yoneda T. (2000). Cell-cell contact between marrow stromal cells and myeloma cells via VCAM-1 and alpha(4)beta(1)-integrin enhances production of osteoclast-stimulating activity. Blood.

[40] Hideshima T., Podar K., Chauhan D., Anderson K.C. (2005). Cytokines and signal transduction. Best Pract. Res. Clin. Haematol..

[41] Gupta D., Treon S.P., Shima Y., Hideshima T., Podar K., Tai Y.T., Lin B., Lentzsch S., Davies F.E., Chauhan D. (2001). Adherence of multiple myeloma cells to bone marrow stromal cells upregulates vascular endothelial growth factor secretion: Therapeutic applications. Leukemia.

[42] Giuliani N., Storti P., Bolzoni M., Palma B.D., Bonomini S. (2011). Angiogenesis and multiple myeloma. Cancer Microenviron..

[43] Terpos E., Kastritis E., Christoulas D., Gkotzamanidou M., Eleutherakis-Papaiakovou E., Kanellias N., Papatheodorou A., Dimopoulos M.A. (2012). Circulating activin-A is elevated in patients with advanced multiple myeloma and correlates with extensive bone involvement and inferior survival; no alterations post-lenalidomide and dexamethasone therapy. Ann. Oncol..

[44] Vallet S., Mukherjee S., Vaghela N., Hideshima T., Fulciniti M., Pozzi S., Santo L., Cirstea D., Patel K., Sohani A.R. (2010). Activin A promotes multiple myeloma-induced osteolysis and is a promising target for myeloma bone disease. Proc. Natl. Acad. Sci. USA.

[45] Berenson J.R., Lichtenstein A., Porter L., Dimopoulos M.A., Bordoni R., George S., Lipton A., Keller A., Ballester O., Kovacs M.J. (1996). Efficacy of pamidronate in reducing skeletal events in patients with advanced multiple myeloma. Myeloma Aredia Study Group. New Engl. J. Med..

[46] Terpos E., Sezer O., Croucher P.I., García-Sanz R., Boccadoro M., San Miguel J., Ashcroft J., Bladé J., Cavo M., Delforge M. (2009). The use of bisphosphonates in multiple myeloma: Recommendations of an expert panel on behalf of the European Myeloma Network. Ann. Oncol..

[47] Raje N., Anderson K.C. (2000). Introduction: The evolving role of bisphosphonate therapy in multiple myeloma. Blood.

[48] Mhaskar R., Kumar A., Miladinovic B., Djulbegovic B. (2017). Bisphosphonates in multiple myeloma: An updated network meta-analysis. Cochrane. Database Syst. Rev..

[49] Major P., Lortholary A., Hon J., Abdi E., Mills G., Menssen H.D., Yunus F., Bell R., Body J., Quebe-Fehling E. (2001). Zoledronic acid is superior to pamidronate in the treatment of hypercalcemia of malignancy: A pooled analysis of two randomized, controlled clinical trials. J. Clin. Oncol..

[50] Kostenuik P.J., Nguyen H.Q., McCabe J., Warmington K.S., Kurahara C., Sun N., Chen C., Li L., Cattley R.C., Van G. (2009). Denosumab, a fully human monoclonal antibody to RANKL, inhibits bone resorption and increases BMD in knock-in mice that express chimeric (murine/human) RANKL. J. Bone Miner. Res..

[51] Raje N., Terpos E., Willenbacher W., Shimizu K., García-Sanz R., Durie B., Legieć W., Krejčí M., Laribi K., Zhu L. (2018). Denosumab versus zoledronic acid in bone disease treatment of newly diagnosed multiple myeloma: An international, double-blind, double-dummy, randomised, controlled, phase 3 study. Lancet Oncol..

[52] Tsourdi E., Langdahl B., Cohen-Solal M., Aubry-Rozier B., Eriksen E.F., Guañabens N., Obermayer-Pietsch B., Ralston S.H., Eastell R., Zillikens M.C. (2017). Discontinuation of Denosumab therapy for osteoporosis: A systematic review and position statement by ECTS. Bone.

[53] von Metzler I., Krebbel H., Hecht M., Manz R.A., Fleissner C., Mieth M., Kaiser M., Jakob C., Sterz J., Kleeberg L. (2007). Bortezomib inhibits human osteoclastogenesis. Leukemia.

[54] Toscani D., Palumbo C., Dalla Palma B., Ferretti M., Bolzoni M., Marchica V., Sena P., Martella E., Mancini C., Ferri V. (2016). The Proteasome Inhibitor Bortezomib Maintains Osteocyte Viability in Multiple Myeloma Patients by Reducing Both Apoptosis and Autophagy: A New Function for Proteasome Inhibitors. J. Bone Miner. Res..

[55] Fulciniti M., Tassone P., Hideshima T., Vallet S., Nanjappa P., Ettenberg S.A., Shen Z., Patel N., Tai Y.T., Chauhan D. (2009). Anti-DKK1 mAb (BHQ880) as a potential therapeutic agent for multiple myeloma. Blood.

[56] Heath D.J., Chantry A.D., Buckle C.H., Coulton L., Shaughnessy J.D., Evans H.R., Snowden J.A., Stover D.R., Vanderkerken K., Croucher P.I. (2009). Inhibiting Dickkopf-1 (Dkk1) removes suppression of bone formation and prevents the development of osteolytic bone disease in multiple myeloma. J. Bone Miner. Res..

[57] Iyer S.P., Beck J.T., Stewart A.K., Shah J., Kelly K.R., Isaacs R., Bilic S., Sen S., Munshi N.C. (2014). A Phase IB multicentre dose-determination study of BHQ880 in combination with anti-myeloma therapy and zoledronic acid in patients with relapsed or refractory multiple myeloma and prior skeletal-related events. Br. J. Haematol..

[58] Delgado-Calle J., Anderson J., Cregor M.D., Condon K.W., Kuhstoss S.A., Plotkin L.I., Bellido T., Roodman G.D. (2017). Genetic deletion of Sost or pharmacological inhibition of sclerostin prevent multiple myeloma-induced bone disease without affecting tumor growth. Leukemia.

[59] McDonald M.M., Reagan M.R., Youlten S.E., Mohanty S.T., Seckinger A., Terry R.L., Pettitt J.A., Simic M.K., Cheng T.L., Morse A. (2017). Inhibiting the osteocyte-specific protein sclerostin increases bone mass and fracture resistance in multiple myeloma. Blood.

[60] Valent P., Akin C., Metcalfe D.D. (2017). Mastocytosis: 2016, pdated WHO classification and novel emerging treatment concepts. Blood.

[61] Lim K.H., Tefferi A., Lasho T.L., Finke C., Patnaik M., Butterfield J.H., McClure R.F., Li C.Y., Pardanani A. (2009). Systemic mastocytosis in 342 consecutive adults: Survival studies and prognostic factors. Blood.

[62] Uzzaman A., Maric I., Noel P., Kettelhut B.V., Metcalfe D., Carter M.C. (2009). Pediatric-onset mastocytosis: A long term clinical follow-up and correlation with bone marrow histopathology. Pediatr. Blood Cancer.

[63] von Bubnoff N., Gorantla S.H., Kancha R.K., Lordick F., Peschel C., Duyster J. (2005). The systemic mastocytosis-specific activating cKit mutation D816V can be inhibited by the tyrosine kinase inhibitor AMN107. Leukemia.

[64] Rossini M., Zanotti R., Bonadonna P., Artuso A., Caruso B., Schena D., Vecchiato D., Bonifacio M., Viapiana O., Gatti D. (2011). Bone mineral density, bone turnover markers and fractures in patients with indolent systemic mastocytosis. Bone.

[65] van der Veer E., van der Goot W., de Monchy J.G., Kluin-Nelemans H.C., van Doormaal J.J. (2012). High prevalence of fractures and osteoporosis in patients with indolent systemic mastocytosis. Allergy.

[66] Seitz S., Barvencik F., Koehne T., Priemel M., Pogoda P., Semler J., Minne H., Pfeiffer M., Zustin J., Püschel K. (2013). Increased osteoblast and osteoclast indices in individuals with systemic mastocytosis. Osteoporos. Int..

[67] Greene L.W., Asadipooya K., Corradi P.F., Akin C. (2016). Endocrine manifestations of systemic mastocytosis in bone. Rev. Endocr. Metab. Disord..

[68] Rossini M., Viapiana O., Adami S., Idolazzi L., Zanotti R., Gatti D. (2015). Rapid skeletal turnover in radiographic mimic of osteopetrosis might be secondary to systemic mastocytosis [corrected]. J. Bone Miner. Res..

[69] Kushnir-Sukhov N.M., Brittain E., Reynolds J.C., Akin C., Metcalfe D.D. (2006). Elevated tryptase levels are associated with greater bone density in a cohort of patients with mastocytosis. Int. Arch. Allergy Immunol..

[70] Theoharides T.C., Boucher W., Spear K. (2002). Serum interleukin-6 reflects disease severity and osteoporosis in mastocytosis patients. Lnt. Arch. Allergy lmmunol..

[71] Dobigny C., Saffar J.L. (1997). H1 and H2 histamine receptors modulate osteoclastic resorption by different pathways: Evidence obtained by using receptor antago nists in a rat synchronized resorption model. J. Cell Physiol..

[72] Biosse-Duplan M., Baroukh B., Dy M., de Vernejoul M.C., Saffar J.L. (2009). Histamine promotes osteoclastogenesis through the differential expression of histamine receptors on osteoclasts and osteoblasts. Am. J. Pathol..

[73] Rossini M., Zanotti R., Viapiana O., Tripi G., Orsolini G., Idolazzi L., Bonadonna P., Schena D., Escribano L., Adami S. (2014). Bone involvement and osteoporosis in mastocytosis. Immunol. Allergy Clin. North Am..

[74] Rossini M., Viapiana O., Zanotti R., Tripi G., Perbellini O., Idolazzi L., Bonifacio M., Adami S., Gatti D. (2015). Dickkopf-1 and sclerostin serum levels in patients with systemic mastocytosis. Calcif. Tissue Int..

[75] Rabenhorst A., Christopeit B., Leja S., Gerbaulet A., Kleiner S., Förster A., Raap U., Wickenhauser C., Hartmann K. (2013). Serum levels of bone cytokines are increased in indolent systemic mastocytosis associated with osteopenia or osteoporosis. J. Allergy Clin. Immunol..

[76] Laroche M., Livideanu C., Paul C., Cantagrel A. (2011). Interferon alpha and pamidronate in osteoporosis with fracture secondary to mastocytosis. Am. J. Med..

[77] Guillaume N., Desoutter J., Chandesris O., Merlusca L., Henry I., Georgin-Lavialle S., Barete S., Hirsch I., Bouredji D., Royer B. (2013). Bone complications of mastocytosis: A link between clinical and biological characteristics. Am. J. Med..

[78] Barete S., Assous N., de Gennes C., Grandpeix C., Feger F., Palmerini F., Dubreuil P., Arock M., Roux C., Launay J.M. (2010). Systemic mastocytosis and bone involvement in a cohort of 75 patients. Ann. Rheum. Dis..

[79] Cundy T., Beneton M.N., Darby A.J., Marshall W.J., Kanis J.A. (1987). Osteopenia in systemic mastocytosis: Natural history and responses to treatment with inhibitors of bone resorption. Bone.

[80] Rossini M., Zanotti R., Viapiana O., Tripi G., Idolazzi L., Biondan M., Orsolini G., Bonadonna P., Adami S., Gatti D. (2014). Zoledronic acid in osteoporosis secondary to mastocytosis. Am. J. Med..

[81] Ali A.S., Lax A.S., Liljeström M., Paakkari I., Ashammakhi N., Kovanen P.T., Konttinen Y.T. (2006). Mast cells in atherosclerosis as a source of the cytokine RANKL. Clin. Chem. Lab Med..

[82] Orsolini G., Gavioli I., Tripi G., Viapiana O., Gatti D., Idolazzi L., Zanotti R., Rossini M. (2017). Denosumab for the Treatment of Mastocytosis-Related Osteoporosis: A Case Series. Calcif. Tissue Int..

[83] Pootrakul P., Hungsprenges S., Fucharoen S., Baylink D., Thompson E., English E., Lee M., Burnell J., Finch C. (1981). Relation between erythropoiesis and bone metabolism in thalassemia. New Engl. J. Med..

[84] Ruggiero L., De Sanctis V. (1998). Multicentre study on prevalence of fractures in transfusion dependent thalassemic patients. J. Pediatr. Endocrinol. Metab..

[85] Poggi M., Sorrentino F., Pugliese P., Smacchia M.P., Daniele C., Equitani F., Terlizzi F., Guitarrini M.R., Monti S., Maffei L. (2016). Longitudinal changes of endocrine and bone disease in adults with β-thalassemia major receiving different iron chelators over 5 years. Ann. Hematol..

[86] Jensen C.E., Tuck S.M., Agnew J.E., Koneru S., Morris R.W., Yardumian A., Prescott E., Hoffbrand A.V., Wonke B. (1998). High prevalence of low bone mass in thalassaemia major. Br. J. Haematol..

[87] Gaudio A., Morabito N., Catalano A., Rapisarda R., Xourafa A., Lasco A. (2019). Pathogenesis of Thalassemia Major-associated Osteoporosis: A Review with Insights from Clinical Experience. J. Clin. Res. Pediatr. Endocrinol..

[88] Fung E.B., Harmatz P.R., Milet M., Coates T.D., Thompson A.A., Ranalli M., Mignaca R., Scher C., Giardina P., Robertson S. (2008). Fracture prevalence and relationship to endocrinopathy in iron overloaded patients with sickle cell disease and thalassemia. Bone.

[89] Vogiatzi M.G., Macklin E.A., Fung E.B., Vichinsky E., Olivieri N., Kwiatkowski J., Cohen A., Neufeld E., Giardina P.J. (2006). Prevalence of fractures among the Thalassemia syndromes in North America. Bone.

[90] Vogiatzi M.G., Macklin E.A., Fung E.B., Cheung A.M., Vichinsky E., Olivieri N., Kirby M., Kwiatkowski J.L., Cunningham M., Holm I.A. (2009). Bone disease in thalassemia: A frequent and still unresolved problem. J. Bone Miner. Res..

[91] Engkakul P., Mahachoklertwattana P., Jaovisidha S., Chuansumrit A., Poomthavorn P., Chitrapazt N., Chuncharunee S. (2013). Unrecognized vertebral fractures in adolescents and young adults with thalassemia syndromes. J. Pediatr. Hematol. Oncol..

[92] Anapliotou M.L., Kastanias I.T., Psara P., Evangelou E.A., Liparaki M., Dimitriou P. (1995). The contribution of hypogonadism to the development of osteoporosis in thalassemia major: New therapeutic approaches. Clin. Endocrinol..

[93] Soliman A.T., El Banna N., Abdel Fattah M., El Zalabani M.M., Ansari B.M. (1998). Bone mineral density in prepubertal children with β-thalassemia: Correlation with growth and hormonal data. Metabolism.

[94] Scacchi M., Danesi L., Cattaneo A., Valassi E., Pecori Giraldi F., Argento C., D’Angelo E., Mirra N., Carnelli V., Zanaboni L. (2008). Bone demineralization in adult thalassaemic patients: Contribution of GH and IGF-I at different skeletal sites. Clin. Endocrinol..

[95] Lasco A., Morabito N., Gaudio A., Crisafulli A., Meo A., Denuzzo G., Frisina N. (2002). Osteoporosis and beta-thalassemia major: Role of the IGF-I/IGFBP-III axis. J. Endocrinol. Invest..

[96] Morabito N., Russo G.T., Gaudio A., Lasco A., Catalano A., Morini E., Franchina F., Maisano D., La Rosa M., Plota M. (2007). The “lively” cytokines network in beta-Thalassemia Major-related osteoporosis. Bone.

[97] Bordat C., Constans A., Bouet O., Blanc I., Trubert C.L., Girot R., Cournot G. (1993). Iron distribution in thalassemic bone by energy-loss spectroscopy and electron spectroscopic imaging. Calcif. Tissue Int..

[98] Chan Y.L., Pang L.M., Chik K.W., Cheng J.C., Li C.K. (2002). Patterns of bone diseases in transfusion-dependent homozygous thalassaemia major: Predominance of osteoporosis and desferrioxamine-induced bone dysplasia. Pediatr. Radiol..

[99] Dandona P., Menon R.K., Houlder S., Thomas M., Hoffbrand A.V., Flynn D.M. (1987). Serum 1,25 dihydroxyvitamin D and osteocalcin concentrations in thalassemia major. Arch. Dis. Child..

[100] Gaudio A., Morabito N., Xourafa A., Currò M., Caccamo D., Ferlazzo N., Macrì I., La Rosa M.A., Meo A., Ientile R. (2010). Role of genetic pattern on bone mineral density in thalassemic patients. Clin. Biochem..

[101] Morabito N., Gaudio A., Lasco A., Atteritano M., Pizzoleo M.A., Cincotta M., La Rosa M., Guarino R., Meo A., Frisina N. (2004). Osteoprotegerin and RANKL in the pathogenesis of thalassemia-induced osteoporosis: New pieces of the puzzle. J. Bone Miner. Res..

[102] Voskaridou E., Christoulas D., Plata E., Bratengeier C., Anastasilakis A.D., Komninaka V., Kaliontzi D., Gkotzamanidou M., Polyzos S.A., Dimopoulou M. (2012). High circulating sclerostin is present in patients with thalassemia-associated osteoporosis and correlates with bone mineral density. Horm. Metab. Res..

[103] Borgna-Pignatti C., Marsella M. (2015). Iron Chelation in Thalassemia Major. Clin. Ther..

[104] Dede A.D., Trovas G., Chronopoulos E., Triantafyllopoulos I.K., Dontas I., Papaioannou N., Tournis S. (2016). Thalassemia-associated osteoporosis: A systematic review on treatment and brief overview of the disease. Osteoporos. Int..

[105] Lasco A., Morabito N., Gaudio A., Buemi M., Wasniewska M., Frisina N. (2001). Effects of hormonal replacement therapy on bone metabolism in young adults with beta-thalassemia major. Osteoporos. Int..

[106] Origa R. (2017). β-Thalassemia. Genet. Med..

[107] Giusti A., Pinto V., Forni G.L., Pilotto A. (2016). Management of beta-thalassemia-associated osteoporosis. Ann. NY Acad. Sci..

[108] Tsartsalis A.N., Lambrou G.I., Tsartsalis D., Savvidis C., Karantza M., Terpos E., Kanaka-Gantenbein C., Chrousos G.P., Kattamis A. (2018). The role of biphosphonates in the management of thalassemia-induced osteoporosis: A systematic review and meta-analysis. Hormones.

[109] Gaudio A., Morabito N., Xourafa A., Macrì I., Meo A., Morgante S., Trifiletti A., Lasco A., Frisina N. (2008). Bisphosphonates in the treatment of thalassemia-associated osteoporosis. J. Endocrinol. Invest..

[110] Voskaridou E., Ntanasis-Stathopoulos I., Papaefstathiou A., Christoulas D., Dimopoulou M., Repa K., Papatheodorou A., Peppa M., Terpos E. (2018). Denosumab in transfusion-dependent thalassemia osteoporosis: A randomized, placebo-controlled, double-blind phase 2b clinical trial. Blood Adv..

[111] Morabito N., Catalano A., Gaudio A., Morini E., Bruno L.M., Basile G., Tsiantouli E., Bellone F., Agostino R.M., Piraino B. (2016). Effects of strontium ranelate on bone mass and bone turnover in women with thalassemia major-related osteoporosis. J. Bone Miner. Metab..

[112] Tournis S., Dede A.D., Savvidis C., Triantafyllopoulos I.K., Kattamis A., Papaioannou N. (2015). Effects of teriparatide retreatment in a patient with β-thalassemia major. Transfusion.

[113] Pinto V.M., Balocco M., Quintino S., Forni G.L. (2019). Sickle cell disease: A review for the internist. Int. Emerg. Med..

[114] Miller R.G., Segal J.B., Ashar B.H., Leung S., Ahmed S., Siddique S., Rice T., Lanzkron S. (2006). High prevalence and correlates of low bone mineral density in young adults with sickle cell disease. Am. J. Hematol..

[115] Garadah T.S., Hassan A.B., Jaradat A.A., Diab D.E., Kalafalla H.O., Kalifa A.K., Sequeira R.P., Alawadi A.H. (2015). Predictors of abnormal bone mass density in adult patients with homozygous sickle-cell disease. Clin. Med. Insights Endocrinol. Diabetes.

[116] Bordbar M.R., Haghpanah S., Zarei T., Dabbaghmanesh M.H., Omrani G.R., Saki F. (2017). Evaluation of bone mineral density in children with sickle-cell anemia and its associated factors in the south of Iran: A case-control study. Arch. Osteoporos..

[117] De Luna G., Ranque B., Courbebaisse M., Ribeil J.A., Khimoud D., Dupeux S., Silvera J., Offredo L., Pouchot J., Arlet J.B. (2018). High bone mineral density in sickle cell disease: Prevalence and characteristics. Bone.

[118] Voskaridou E., Stoupa E., Antoniadou L., Premetis E., Konstantopoulos K., Papassotiriou I., Terpos E. (2006). Osteoporosis and osteosclerosis in sickle cell/beta-thalassemia: The role of the RANKL/osteoprotegerin axis. Haematologica.

[119] Sadat-Ali M., Al-Elq A., Sultan O., Al-Turki H. (2008). Secondary osteoporosis due to sickle cell anemia: Do sex steroids play a role?. Indian J. Med. Sci..

[120] Boettger P.C., Knupp C.L., Liles D.K., Walker K. (2017). Vitamin D Deficiency in Adult Sickle Cell Patients. J. Natl. Med. Assoc..

[121] Paschou S.A., Anagnostis P., Karras S., Annweiler C., Vakalopoulou S., Garipidou V., Goulis D.G. (2014). Bone mineral density in men and children with haemophilia A and B: A systematic review and meta-analysis. Osteoporos. Int..

[122] Rodriguez-Merchan E.C., Valentino L.A. (2019). Increased bone resorption in hemophilia. Blood Rev..

[123] Tang G.H., Norris E., Petrucci J., James P.D., Lee A., Poon M.C., Floros G., Boma-Fischer L., Teitel J., Nisenbaum R. (2019). Bone health in symptomatic carriers of haemophilia A: A protocol for a multicentre prospective matched-cohort study. BMJ Open.

[124] Mansouritorghabeh H., Rezaieyazdi Z. (2017). Bone Density Status in Bleeding Disorders: Where Are We and What Needs to be Done?. J. Bone Metab..

[125] Gerstner G., Damiano M.L., Tom A., Worman C., Schultz W., Recht M., Stopeck A.T. (2009). Prevalence and risk factors associated with decreased bone mineral density in patients with haemophilia. Haemophilia.

[126] Iorio A., Fabbriciani G., Marcucci M., Brozzetti M., Filipponi P. (2010). Bone mineral density in haemophilia patients. A meta-analysis. Thromb. Haemost..

[127] Hoots W.K., Rodriguez N., Boggio L., Valentino L.A. (2007). Pathogenesis of haemophilic synovitis: Clinical aspects. Haemophilia.

[128] Forsyth A.L., Quon D.V., Konkle B.A. (2011). Role of exercise and physical activity on haemophilic arthropathy, fall prevention and osteoporosis. Haemophilia.

[129] Anagnostis P., Vakalopoulou S., Slavakis A., Charizopoulou M., Kazantzidou E., Chrysopoulou T., Vyzantiadis T.A., Moka E., Agapidou A., Garipidou V. (2012). Reduced bone mineral density in patients with haemophilia A and B in Northern Greece. Thromb. Haemost..

[130] Kempton C.L., Antoniucci D.M., Rodriguez-Merchan E.C. (2015). Bone health in persons with haemophilia. Haemophilia.

[131] Anagnostis P., Vyzantiadis T.A., Charizopoulou M., Adamidou F., Karras S., Goulis D.G., Karagiannis A., Garipidou V., Vakalopoulou S. (2013). The effect of monthly ibandronate on bone mineral density and bone turnover markers in patients with haemophilia A and B and increased risk for fracture. Thromb. Haemost..

[132] Ghosh K., Shetty S. (2012). Bone health in persons with haemophilia: A review. Eur. J. Haematol..

